# A Larger Membrane Area Increases Cytokine Removal in Polymethyl Methacrylate Hemofilters

**DOI:** 10.3390/membranes12080811

**Published:** 2022-08-22

**Authors:** Tomoyuki Nakamura, Kazuhiro Moriyama, Naohide Kuriyama, Yoshitaka Hara, Satoshi Komatsu, Takahiro Kawaji, Yu Kato, Takuma Ishihara, Ayumi Shintani, Osamu Nishida

**Affiliations:** 1 Department of Anesthesiology and Critical Care Medicine, Fujita Health University School of Medicine, Toyoake 470-1192, Japan; 2 Laboratory for Immune Response and Regulatory Medicine, Fujita Health University School of Medicine, Toyoake 470-1192, Japan; 3 Innovative and Clinical Research Promotion Center, Gifu University Hospital, Gifu 501-1193, Japan; 4 Department of Medical Statistics, Graduate School of Medicine, Osaka Metropolitan University, 1-4-3 Asahimachi, Abeno-ku, Osaka 545-8585, Japan

**Keywords:** adsorption, blood purification, clearance, hemofilters, HMGB-1, hydrophobic binding, IL-6, membrane area, polymethyl methacrylate

## Abstract

Blood purification is performed to control cytokines in critically ill patients. The relationship between the clearance (CL) and the membrane area during adsorption is not clear. We hypothesized that the CL increases with the hydrophobic area when hydrophobic binding contributes to cytokine adsorption. We investigated the relationship between the hemofilter membrane area and the CL of the high mobility group box 1 protein (HMGB-1) and interleukin-6 (IL-6). We performed experimental hemofiltration in vitro using polymethyl methacrylate membranes CH-1.8W (1.8 m^2^) and CH-1.0N (1.0 m^2^), as well as polysulfone membrane NV-18X (1.8 m^2^). After adding 100 mg of HMGB1 or 10 μg of IL-6 into the test solution, experimental hemofiltration was conducted for 360 min in a closed-loop circulation system, and the same amount of HMGB1 and IL-6 was added after 180 min. With CH-1.8W and CH-1.0N, both HMGB-1 and IL-6 showed a rapid concentration decrease of more than 70% at 180 min and 360 min after the re-addition. At 15 min, the CL of HMGB-1 was CH-1.8W: 28.4 and CH-1.0N: 19.8, and that of IL-6 was CH-1.8W: 41.1 and CH-1.0N: 25.4. CH-1.8W and CH-1.0N removed HMGB1 and IL-6 by adsorption and CH-1.8W was superior in CL, which increased with a greater membrane area.

## 1. Introduction

Blood purification plays an important role in the treatment of patients who are severely ill, including those with sepsis, and is often performed not only as renal replacement therapy, but also for the modulation of cytokines and other humoral mediators [1,2,3]. Sepsis occurs when there is organ damage caused by an uncontrolled host immune response that has been triggered by infection [4] and its pathogenesis, hypercytokinemia, includes interleukin-1 (IL-1), interleukin-6 (IL-6), tumor necrosis factor, and high mobility group box 1 protein (HMGB-1), which is a major inflammatory cytokine [5]. IL-6 is a cytokine released by the immune cells and plays a role in the systemic inflammatory changes caused by infection or tissue injury [6]. Recently, IL-6 produced by the marginal zone B cells in animal experiments was found to be a factor that promoted the development of sepsis. In these experiments, an improvement in mortality from sepsis by administering IL-6 antibodies has been reported [7]. Several studies have also reported that the serum IL-6 concentration is associated with disease severity [8], organ dysfunction [9], and overall mortality among patients with sepsis, burn and trauma injuries, and cardiovascular diseases, as well as those undergoing hemodialysis [10,11,12,13]. HMGB-1 is one of the DAMPs (damage associated molecular patterns) released from injured tissues into the bloodstream that metastasizes and amplifies inflammation. Wang et al. reported that HMGB-1 was a late mediator of endotoxin lethality and that the serum levels of HMGB-1 were increased significantly in the septic patients who did not survive compared with the survivors [14]. Hence, HMGB-1 has been studied as a key mediator of sepsis. In clinical settings, the plasma levels of HMGB-1 were associated with the severity and mortality attributed to sepsis and correlated with RIPK3 (receptor interacting protein kinase 3) and MLKL (mixed lineage kinase domain-like protein), suggesting an association of HMGB-1 with necroptosis [15].

In our previous investigation of HMGB-1, we found that the efficiency of the removal differed based on the membrane material of the hemofilter [16]; although the upper limit of the filtration clearance (CL) was the filtrate flow rate, HMGB-1 with a molecular weight of 30 kD was removed with a several times greater efficiency than the filtration clearance of the small molecular weight creatinine. This may be explained by the principle of adsorption [17]. In cases of continuous kidney replacement therapy, where the intensity of blood purification is low, the effect of the membrane area on the CL due to filtration or dialysis is negligible [1]. However, the relationship between the CL and the membrane area in cases of adsorption remains unknown.

Hydrophilic binding, such as ionic binding, and hydrophobic binding are the typical adsorption principles of hemofilters. Hydrophilic bonds involve polar molecules with negatively charged oxygen and positively charged hydrogen ions. Typical hydrophobic substances in vivo are membrane proteins, while hydrophilic substances include proteins dissolved in blood, which are determined by the number and steric configuration of the hydrophilic and hydrophobic groups. On the one hand, the ionic binding is affected by the charge of the target substance (isoelectric point in the case of proteins), and it has been described as an adsorption mechanism for acrylonitrile-co-methallyl sulfonate surface-treated (AN69ST) materials [18]. On the other hand, hydrophobic binding is affected by the molecular weight of the target substance and the hydrophobic area (adsorption site) of the membrane. However, the effect of the membrane area of the hydrophobic binding membrane on cytokines has not been evaluated. While *p*-methyl methacrylate (PMMA) acrylic resin is hydrophobic [19], it is not clear whether the PMMA membrane hemofilter adsorbs cytokines via hydrophobic binding [1].

We hypothesized that, if hydrophobic binding contributes to cytokine adsorption, the CL will increase in parallel with an increase in the hydrophobic area. We investigated the relationship between the hemofilter membrane area and the CL of HMGB-1 and IL-6 in vitro using PMMA membranes with different membrane areas using a polysulfone membrane as a non-adsorptive reference.

## 2. Materials and Methods

**Figure 1:**
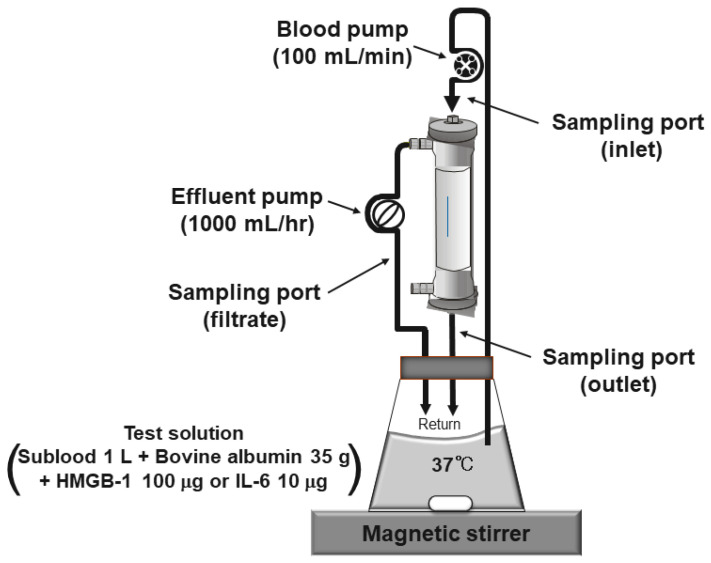
Diagram of the in vitro experimental setup. This is a recirculation model for testing the adsorption mechanism. Only when the adsorption occurred, did the concentration of HMGB-1 or IL-6 at the inlet side decrease.

The in vitro, closed, experimental system consisted of a solution reservoir, hemofilter, and fully automated blood purification machine (TR525, Toray Medical Co., Tokyo, Japan), as shown in Figure 1. The hollow fiber hemofilters investigated were CH-1.8W (Toray Ind., Tokyo, Japan; polymethylmethacrylate: PMMA) with a large membrane area of 1.8 m^2^, CH-1.0N (Toray, Tokyo, Japan; PMMA) with a normal membrane area of 1.0 m^2^, and NV-18X (Toray, Tokyo, Japan; polysulfone: PS) with a large membrane area of 1.8 m^2^. In the evaluation of CH-1.8W, CH-1.0N was used for the membrane area comparison and NV-18X was used as the control membrane. The test solution was prepared by dissolving 35 g of bovine serum albumin (MW 66 kDa; Fuji Film Wako Pure Chemical Co., Osaka, Japan) in 1000 mL of the substitution fluid for hemofiltration Sublood BSG^®^ (Fuso Pharmaceutical Ind., Osaka, Japan), adding 100 μg of HMGB-1 (Shino-Test Co., Sagamihara, Japan) or 10 μg of IL-6 (Kamakura Techno Science, Kamakura, Japan), and stirring the mixture thoroughly with a magnetic stirrer. The circuit containing the hemofiltration membrane was primed with a saline solution and extruded slowly with the above test solution, and then the priming volume was discarded to make a closed circuit. The test solution was pumped from the solution reservoir to a hemofilter at a solution flow rate (Qb) of 100 mL/min and returned to the same reservoir. The ultrafiltrate was pumped in the post-dilution mode at a filtrate flow rate (Qf) of 1000 mL/h (16.7 mL/min) and returned to the reservoir in a closed-loop circulation system. The in-flask concentration decreased only when cytokines were removed by adsorption in this system. The experimental hemofiltration was conducted for 360 min at 37 °C. To confirm the adsorption saturation limit of the membranes, 100 μg of HMGB-1 or 10 μg of IL-6 was added again after 180 min; the mixture was circulated for 5 min to make the solution concentration uniform and then evaluated for a period of 185 min to 360 min. The samples for laboratory analyses were taken from both the inlet and outlet of the filter and from the filtrate port. The in-flask concentration was the same as the inlet concentration. The point at which the test solution was circulated for 3 min to equalize the concentration in the circuit was set at 0 min. The samples were collected at 0, 5, 15, 30, 45, 60, 90, 120, and 180 min. After adding HMGB-1 or IL-6 again after 180 min, the samples were collected at 185, 195, 210, 225, 240, 270, 300, and 360 min. Over time, the clearances were calculated using the following formula:

Solution clearance (mL/min): CL = (CBi − CBo)/CBi × (Qb − Qf) + Qf

Filtrate clearance (mL/min): CLf = Cf/CBi × Qf

Adsorption clearance (mL/min): CLa = CL − CLf

where CBi: solute concentration at the inlet side, CBo: solute concentration at the outlet side, Cf: solute concentration at the filtrate side, Qb: solution flow (applied to blood flow in clinical practice), and Qf: filtrate flow.

To evaluate the effects of time from the addition (or re-addition), the number of times added, interaction between time and number of times added, type of hemofilter, and inlet concentration on the CL, we used a linear mixed effects model with the intercepts being random effects. The modifying effect of the inlet concentration on the association between the type of hemofilter and the CL was assessed using separate models after the initial addition and re-addition, each including a non-linear term and an interaction term between the type of hemofilter and the inlet concentration. The association of the CL was confirmed using an F-test on the regression coefficients obtained from the mixed-effect model. The statistical significance was set at a two-sided *p*-value of <0.05. All of the statistical analyses were performed using R version 4.1.1 patched (http://www.r-project.org, accessed on 1 August 2022).

## 3. Results

**Figure 2:**
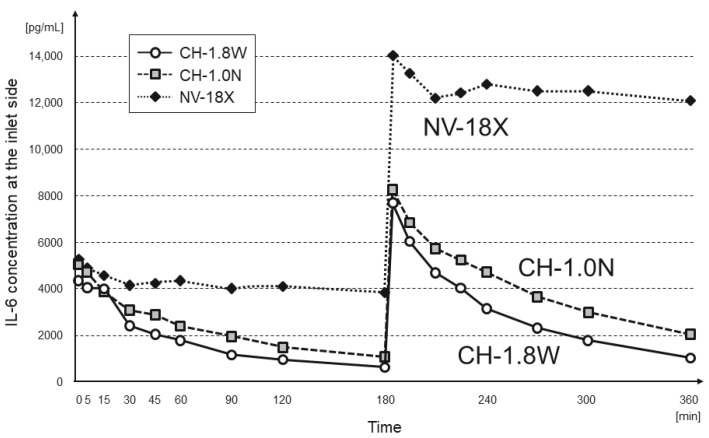
Change in the IL-6 concentration at the inlet side (n = 3 each, mean).

**Figure 3:**
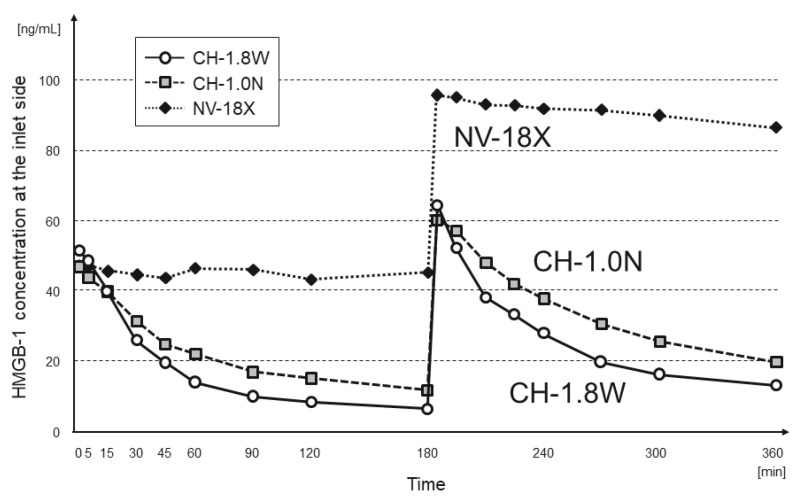
Change in the HMGB-1 concentration at the inlet side (n = 3 each, mean).

The changes in the IL-6 and HMGB-1 concentrations at the inlet of each hemofilter are shown in Figure 2 and Figure 3, respectively. In CH-1.8W and CH-1.0N, both IL-6 and HMGB-1 showed a rapid decrease in concentration of more than 70% at 180 min. Even after re-addition, there was a decrease in the concentration of approximately 70% at 360 min.

In NV-18X, the inlet concentration decreased up to 15 min after addition and then remained almost unchanged until 180 min. After the re-addition, the concentration remained the same after the added amount was increased.

**Figure 4:**
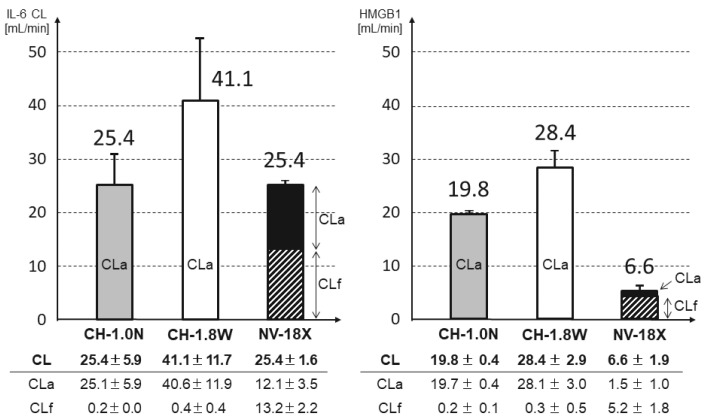
Comparison of the CL (at 15 min). CL: solution clearance, CLa: adsorption clearance, CLf: filtrate clearance. CL = CLa + CLf. The shaded area represents the CLf. In CH-1.0N and CH-1.8W, the CLf is too small to be observed (n = 3 each, mean ± SD).

At 15 min, the IL-6 CL was CH-1.8W: 4 1.1 mL/min, CH-1.0N: 2 5.4 mL/min, and NV-18X: 25.4 mL/min and the HMGB-1 CL was CH-1.8W: 28.4 mL/min, CH-1.0N: 19.8 mL/min, and NV-18X: 6.6 mL/min (Figure 4). With CH-1.8W and CH-1.0N, the CL of both IL-6 and HMGB-1 consisted almost completely of adsorption and increased with the increasing membrane area.

**Figure 5:**
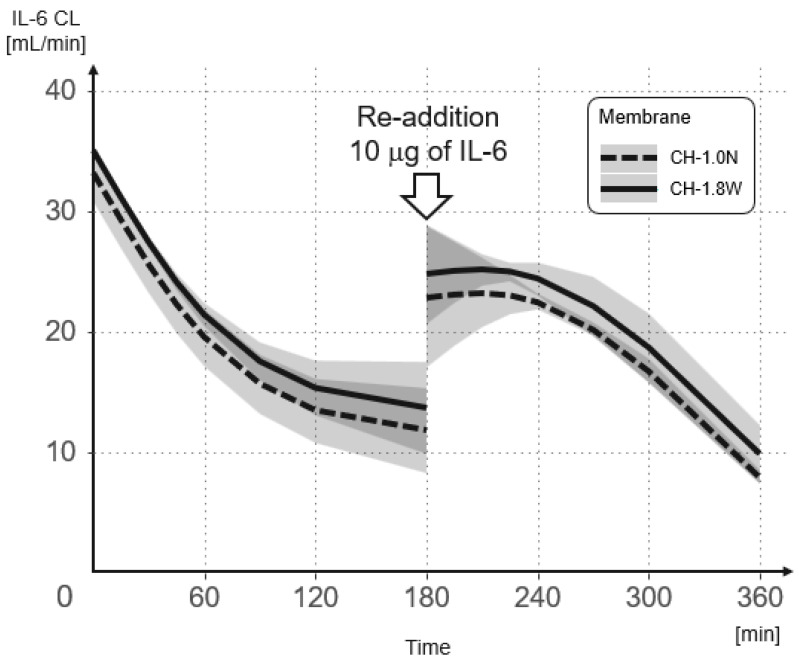
Change in the IL-6 CL over time when using CH-1.0N and CH-1.8W. IL-6 was added again after 180 min to confirm the adsorption saturation limit of the membranes.

The results of the mixed-effects model analysis and the predicted values of change in the IL-6 CL over time for each hemofilter from the initial addition and re-addition are shown in Figure 5 and Table 1. After the re-addition, the IL-6 CL was, on average, 8.879 mL/min lower than after the initial addition (*p* = 0.005). The IL-6 CL decreased by an average of 0.08 mL/min per minute (*p* = 0.001). Furthermore, the IL-6 CL in CH-1.8W was significantly higher than the IL-6 CL in CH-1.0N (3.443) (*p* = 0.016). For every 1 pg/mL increase in concentration, the IL-6 CL increased by an average of 0.002 mL/min (*p* = 0.023). A significant interaction was observed between the number of additions and time (*p* = 0.023).

**Figure 6:**
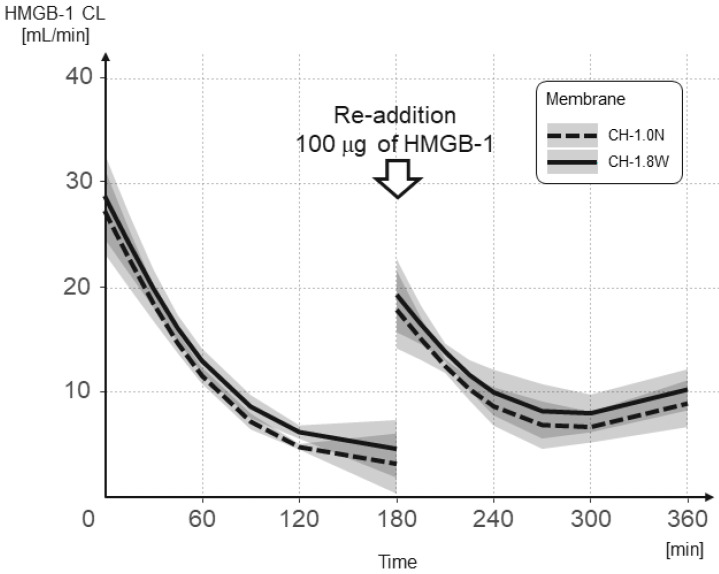
Change in the HMGB-1 CL over time when using CH-1.0N and CH-1.8W. HMGB-1 was added again after 180 min to confirm the adsorption saturation limit of the membranes.

The results of the mixed-effects model analysis and the predicted values of change in the HMGB-1 CL over time for each hemofilter from the initial addition and re-addition are shown in Figure 6 and Table 2. After the re-addition, the HMGB-1 CL was, on average, 13.309 mL/min lower than after the initial addition (*p* < 0.001). The HMGB-1 CL decreased by an average of 0.075 mL/min per minute (*p* < 0.001). The HMGB-1 CL in CH-1.8W was significantly higher than the HMGB-1 CL in CH-1.0N (2.923) (*p* = 0.006). For every 1 ng/mL increase in concentration, the HMGB-1 CL increased by an average of 0.287 mL/min (*p* < 0.001). A significant interaction was observed between the number of additions and time (*p* < 0.001).

**Figure 7:**
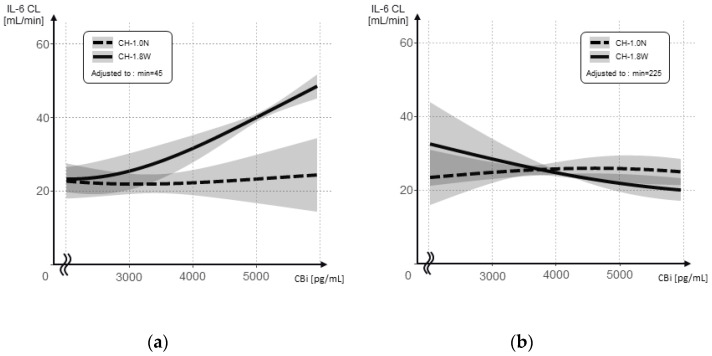
IL-6 CL at different IL-6 concentrations at the inlet side (CBi) when using CH-1.0N and CH-1.8W. (**a**) After the initial addition; (**b**) after the re-addition.

The relationship between the inlet concentration and the IL-6 CL is shown separately after the initial addition and re-addition for each hemofilter (Figure 7a,b). The interaction between the inlet concentration and hemofilter type was significant, after both the initial addition and the following re-addition (*p* = 0.007 and *p* = 0.010, respectively); however, the respective associations showed the opposite results. The CL was higher for CH-1.8W at higher inlet concentrations after the initial addition, whereas after the re-addition, the CL decreased at higher inlet concentrations and remained flat at approximately 20 mL/min.

The results of the linear mixed model evaluating the association between the IL-6 CL and the inlet concentrations are shown after the initial addition and the following re-addition (Table 3). After the initial addition, for every 1 pg/mL increase in concentration, the IL-6 CL increased by an average of 0.005 mL/min (*p* = 0.001). After the re-addition, the IL-6 CL decreased, on average, by 0.002 mL/min for each 1 pg/mL increase in concentration (*p* = 0.049). After re-administration, the IL-6 CL decreased by an average of 0.121 mL/min (*p* = 0.001).

**Figure 8:**
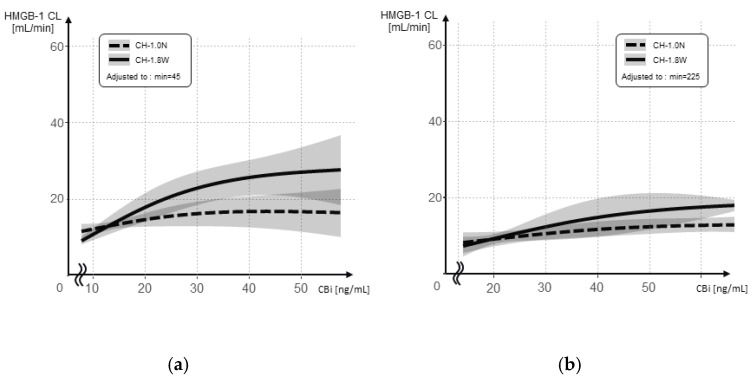
The HMGB-1 CL at different HMGB-1 concentrations at the inlet side (CBi) when using CH-1.0N and CH-1.8W. (**a**) After the initial addition; (**b**) after the re-addition.

The relationship between the inlet concentration and the HMGB-1 CL is shown separately after the initial addition and re-addition for each hemofilter (Figure 8a,b). The interaction between the inlet concentration and hemofilter type was significant after the initial addition (*p* = 0.003).

The results of the evaluation of the association between the HMGB-1 CL and the inlet concentrations using a linear mixed model are shown in Table 4, after the initial addition and the following re-addition. After the initial addition, the HMGB-1 CL decreased by an average of 0.069 mL/min (*p* = 0.001).

## 4. Discussion

In recent years, the control of humoral mediators such as cytokines, using the principle of adsorption, has attracted attention in blood purification therapy for sepsis [20]. Membranes with a high adsorption capacity include AN69ST, oXiris, and PMMA [21]. PMMA and AN69ST membranes have been used extensively in Japan, and many treatment experiences, especially for sepsis, have been reported [22,23,24]. However, there have been no direct reports indicating that the removal of IL-6 or HMGB-1 improved sepsis mortality.

PMMA membranes have a homogenous symmetric structure and may exhibit adsorption capacity owing to hydrophobic binding, not only on the hollow fiber inner surface, but also on the entire membrane, including the bulk layer [25]. The diameter of the pores is designed to be 6.6–7.0 nm, and IL-6, with a radius of 2 nm, is also expected to be adsorbed efficiently on the inner surface of the pore.

During filtration, water and other substances move from the blood to the filtrate side through the pores. If the pore size is sufficiently large to allow the substances to pass through, removal by filtration is expected according to the filtration flow rate (1000 mL/h = 17 mL/min). However, in the present study, there was little removal of both IL-6 and HMGB1 with filtration through the CH-1.0N and CH-1.8W PMMA membranes (Figure 4). This is because most of the substance is adsorbed when passing through the hollow fiber inner surface and bulk layer and, therefore, does not enter the filtrate. This difference in removal capacity according to the membrane area was also observed in the comparison of 15-minute values: the IL-6 CL was approximately 1.6-fold larger and the HMGB-1 CL was approximately 1.4-fold larger when the membrane area was 1.8-fold larger. Although the CL did not increase at the same ratio as the increase in membrane area, it increased with increasing membrane area, indicating that a larger membrane area increases the cytokine removal capacity.

Matsumura et al. devised a method using two PMMA membranes with a large membrane area of 2.1 mm^2^ as enhanced-intensity PMMA-CHDF and used them clinically. They reported that the IL-6 removal ability was superior, and that the mortality rate was also reduced compared with the normal CHDF group that used a 1.0 m^2^ PMMA membrane [26]. As the procedure was performed using two consoles, the efficiency of dialysis and filtration was doubled; however, the increased membrane area may also have had a significant impact.

The increase in the concentration before and after the re-addition of IL-6 was smaller in CH-1.8W and CH-1.0N than in NV-18X. As mentioned above, this is because NV-18X has a low level of removal, whereas CH-1.8W and CH-1.0N continue to be adsorbed and removed for 5 min until the next measurement after the re-addition.

The CL of CH-1.8W and CH-1.0N decreased with time for both IL-6 and HMGB1, but showed a significant increase in CL after the re-addition. This indicates that the adsorption capacity of the membrane did not reach saturation. Compared with the CL at the time of the initial addition, the CL after the re-addition was low, and while the CL decreased with time, it still maintained a sufficient CL. This may have been because of the main adsorption site during the initial addition being the hollow fiber lumen, whereas the adsorption site after the re-addition was the bulk layer.

Figure 6 shows the relationship between the inlet concentration and the IL-6 CL for CH-1.8W and CH-1.0N, without the time factor. In CH-1.8W, after the re-addition, the IL-6 CL appears to be lower at higher inlet concentrations. However, in the actual data, the IL-6 CL in CH-1.8W was higher than in CH-1.0N, while the inlet concentration was high (180–225 min) even after the re-addition (Supplement Table S1). After the re-addition, the inlet concentration was lower in CH-1.8W at all the measurement points, that is, a greater amount of IL-6 was removed. As the substance concentration was lower, the removal efficiency also decreased because the frequency of contact with the adsorption surface was lower. In CH-1.8W, the inlet concentration dropped sharply after 300 min. On the other hand, the CL is calculated from the ratio of the inlet and outlet concentrations, and while an accurate clearance is obtained when the target substance concentration is sufficiently high, a small change is reflected in the clearance, to a large extent, when the target substance concentration is low. The fact that the IL-6 CL appears to be lower at higher inlet concentrations in CH-1.8W after the re-addition may have been because of these errors.

The IL-6 CL after the re-addition was approximately 20 mL/min without the time factor. Although little filtration removal was observed in this study (Figure 4), both showed IL-6 CLs with a filtration flow rate of approximately 17 mL/min, suggesting that adsorption occurs mainly in the bulk layer rather than in the inner surface of the hollow fiber. As adsorption in the bulk layer is considered to occur as the filtration passes through the pores, the efficiency of adsorption in the bulk layer approximates the filtration efficiency. The reason CH-1.8W showed a high IL-6 CL according to the inlet concentration after the initial addition may have been because of CH-1.8W having a sufficiently large membrane area; thus, a high percentage of adsorption was also observed in the inner surface of the hollow fiber at the initial addition. After the adsorption sites on the hollow fiber inner surface reached saturation, adsorption would occur in the bulk layer, so the IL-6 CL was considered to be equivalent to that of CH-1.0N according to a filtration flow rate of 17 mL/min. The nature and capacity of adsorption differs between the inner surface of the hollow fiber and the bulk layer, suggesting that they act as if they are different membranes. The hollow fiber lumen surface is 1.8 m^2^ or 1.0 m^2^, as described in the manufacturer’s instructions. On the other hand, just as the intestinal tract has a huge surface area because of its numerous microvilli, the surface area of the bulk layer is considered to have a huge surface area compared with the hollow fiber lumen surface.

The added amount of 10 μg of IL-6 used in this study corresponds to the amount of IL-6 in the total circulating blood when a patient, weighing 70 kg and with a circulating blood volume of 4.6 L, develops an IL-6 hypercytokinemia of approximately 2170 pg/mL. At this amount, the adsorption sites on the hollow fiber inner surface reached saturation even at CH-1.8W; however, the adsorption capacity in the bulk layer did not reach saturation even at CH-1.0N and still had sufficient adsorption capacity. Although not considered in this study, a larger membrane area may have a higher adsorption capacity, as the bulk layer volume is inevitably larger, and the surface area of the hydrophobic material in the pores is larger. Protein adsorption on PMMA membranes is reported to be via an occlusion into pores of the membrane, which is dependent on the filtration and, with a larger membrane area, a larger surface area is adsorbed [27]. When dialysis is used, an even higher adsorption capacity in the bulk layer may be expected because of internal filtration. The present experiment was a simple experimental system comprising additional filtration. However, in a clinical continuous hemodiafiltration practice, there is dialysate flowing in the opposite direction to the blood on the outside of the hollow fiber, and even though the flow rate is lower than that in conventional hemodialysis, a significant internal filtration effect is expected. Therefore, the diffusion, filtration, and adsorption clearances may be even greater. Usually, the circuit exchange for blood purification is performed every day and, considering the high Il-6 CL at the time of the initial addition, a larger membrane area may be advantageous when selecting PMMA membranes.

There were several limitations to this study. First, this was an in vitro study in which bovine albumin was added to a phosphate buffer solution, which has different properties from blood, which contains many other components, and may differ from the adsorption CL in actual clinical practice. However, because it was a simple experimental system, the adsorption characteristics of HMGB-1 and IL-6 on the membrane itself could be evaluated more accurately. Second, both CH-1.0N and CH-1.8W showed a CL reuptake after a single re-addition, indicating that the CL had not reached saturation. However, we could not indicate how many re-additions were required to reach saturation. In our previous multiple addition experiment on an AN69ST membrane, saturation was not reached even after seven re-additions of 100 mcg of HMGB-1 [17]. The adsorption in the bulk layer of the PMMA membrane was significant, and it may have a comparably large adsorption capacity. Third, only HMGB-1 and IL-6 were examined in this study. When the adsorption principle is ionic bonding, the adsorption capacity depends on the charge of the substance to be adsorbed. Meanwhile, the CL increased as the membrane area increased, suggesting that the PMMA membrane hydrophobicity was involved. However, this experiment did not directly demonstrate the presence of any hydrophobic binding between the PMMA membranes and cytokines. Fourth, the hollow fiber inner diameters were different; that is, CH-1.8W: 240 μm and CH-1.0N: 200 μm. For a more accurate comparison of the effect of an increased membrane area, CH-0.6W or CH-1.3W, which have the same hollow fiber inner diameter as CH-1.8W, should be considered and, in addition, further studies are needed.

## 5. Conclusions

The removal characteristics of different membrane materials, membrane areas, and substances to be removed were investigated, and we found that IL-6 and HMGB-1 were removed mainly according to the principle of adsorption in hemofiltration using PMMA membranes, and their removal capacity increased in parallel with an increase in the membrane area. The CL increased as the membrane area increased, suggesting that hydrophobic bonding may be involved in cytokine adsorption, which may allow for the removal of many other cytokines in a non-specific manner. Larger membrane areas may be effective in controlling pathological conditions and in disease control when PMMA membranes are used for blood purification with the intention of controlling mediators.

## Figures and Tables

**Table 1:** The relationship between each parameter, the number of additions, time, hemofilter type, and IL-6 concentration at the inlet side (CBi), and the interaction between the number of additions and CBi, and with the IL-6 CL as an objective variable, using a linear mixed model.

	Coefficient (β)	95%LCI	95%UCI	*p*-Value
Number of additions	−8.879	−14.61	−1.930	0.005 *
Time	−0.080	−0.129	−0.038	0.001 *
Hemofilter type	3.443	0.524	6.047	0.016 *
IL-6 concentration at the inlet side	0.002	0.000	0.003	0.023 *
Interaction between number of additions and time	0.058	0.006	0.104	0.023 *

*: *p*-value of <0.05 was considered as significant.

**Table 2:** The relationship between each parameter, the number of additions, time, hemofilter type, and HMGB-1 concentration at the inlet side (CBi), and interaction between the number of additions and CBi, and with the HMGB-1 CL as an objective variable, using a linear mixed model.

	Coefficient (β)	95%LCI	95%UCI	*p*-Value
Number of additions	−13.309	−16.784	−9.431	<0.001 *
Time	−0.075	−0.112	−0.042	<0.001 *
Hemofilter type	2.923	0.831	4.886	0.006 *
HMGB-1 concentration at the inlet side	0.287	0.148	0.401	<0.001 *
Interaction between number of additions and time	0.099	0.063	0.134	<0.001 *

*: *p*-value of <0.05 was considered as significant.

**Table 3:** The relationship between each parameter, the IL-6 concentration at the inlet side (CBi), hemofilter type, and time, and the interaction between the CBi and hemofilter type, with the IL-6 CL as an objective variable, using a linear mixed model. *: *p*-value of <0.05 was considered as significant.

	After the Initial Addition	Coefficient (β)	95%LCI	95%UCI	*p*-Value
(a)	IL-6 concentration at the inlet side (CBi)	0.005	0.002	0.007	0.001 *
	Hemofilter type	−0.201	−5.807	5.169	0.944
	Time	−0.001	−0.049	0.043	0.980
	Interaction between CBi and hemofilter type	0.003	0.001	0.005	0.007 *
	**After the Re-Addition**	**Coefficient (** **β)**	**95%LCI**	**95%UCI**	***p*** **-Value**
(b)	IL-6 concentration at the inlet side (CBi)	−0.002	−0.004	0.000	0.049 *
	Hemofilter type	−10.677	−18.834	−2.520	0.017
	Time	−0.121	−0.187	−0.055	0.001 *
	Interaction between CBi and hemofilter type	0.002	0.001	0.004	0.010 *

**Table 4:** The relationship between each parameter, the HMGB-1 concentration at the inlet side (CBi), hemofilter type, and time, and the interaction between the CBi and hemofilter type, with the HMGB-1 CL as an objective variable, using a linear mixed model. *: *p*-value of <0.05 was considered as significant.

	After the Initial Addition	Coefficient (β)	95%LCI	95%UCI	*p*-Value
(a)	HMGB-1 concentration at the inlet side (CBi)	0.166	−0.053	0.351	0.102
	Hemofilter type	−3.452	−8.497	1.206	0.175
	Time	−0.069	−0.110	−0.034	0.001 *
	Interaction between CBi and hemofilter type	0.269	0.108	0.441	0.003 *
	**After the Re-Addition**	**Coefficient (** **β)**	**95%LCI**	**95%UCI**	***p*** **-Value**
(b)	HMGB-1 concentration at the inlet side (CBi)	0.044	−0.125	0.246	0.623
	Hemofilter type	−2.951	−9.960	4.057	0.430
	Time	−0.020	−0.062	0.027	0.361
	Interaction between CBi and hemofilter type	0.121	−0.044	0.286	0.173

## Data Availability

Not applicable.

## Supplementary Materials

The following supporting information can be downloaded at: https://www.mdpi.com/article/10.3390/membranes12080811/s1, Supplemental Table S1. The concentration at the inlet side (CBi) and the change in IL-6 CL over time when using CH-1.0N and CH-1.8W. IL-6 was added again after 180 min to confirm the adsorption saturation limit of the membranes.
