# Supplementary material for: A Larger Membrane Area Increases Cytokine Removal in Polymethyl Methacrylate Hemofilters
Source: Membranes (Basel). 2022 Aug 22;12(8):811. doi: 10.3390/membranes12080811 (PMC9413121; doi:10.3390/membranes12080811)
Supplement: Supplementary file 1 [file membranes-12-00811-s001.zip › membranes-1871220-supplementary.pdf]

	Time [min]	5	15	30	45	60	90	120	180	185	195	210	225	240	270	300	360
**CH-1.8W**	CBi [pg/mL]	4098.0	4052.3	2463.7	2090.3	1812.1	1183.3	984.7	650.4	7717.5	6082.2	4738.0	4076.3	3205.8	2362.1	1808.1	1074.5
	CL [mL/min]	34.1	41.1	25.9	19.8	24.5	16.8	14.9	13.1	29.1	25.3	23.8	26.3	25.4	20.7	15.2	2.8
**CH-1.0N**	CBi [pg/mL]	4756.8	3906.2	3134.9	2930.1	2441.0	1960.9	1496.5	1101.1	8303.0	6886.6	5769.1	5288.7	4769.0	3687.7	3011.2	2095.9
	CL [mL/min]	31.3	25.4	18.4	25.4	17.9	20.3	11.9	12.2	23.4	19.4	19.0	21.8	27.2	19.5	20.4	14.9

Supplemental Table S1. The concentration at the inlet side (CBi) and the change in IL-6 CL over time when using CH-1.0N and CH-1.8W. IL-6 was added again after 180 min to confirm the adsorption saturation limit of the membranes.
